# Zosteriform Lichen Planus on the Trunk: A Case Report of a Rare Clinical Entity

**DOI:** 10.7759/cureus.22867

**Published:** 2022-03-05

**Authors:** Fedaa Andijani

**Affiliations:** 1 Department of Dermatology, King Abdulaziz University Hospital, Jeddah, SAU

**Keywords:** pigmented skin lesion, blaschko’s line, linear dermatosis, zosteriform, lichen planus

## Abstract

Zosteriform lichen planus is a variant of cutaneous lichen planus that may develop at the site of healed herpes zoster or may evolve spontaneously with no previous history of herpes zoster or varicella-zoster virus infection. Lichen planus is an immune-mediated disorder that affects the skin and mucous membrane. Nonetheless, its exact etiology remains unclear. The lesion consists of polygonal, pruritic, flat-topped papules that may coalesce to form a plaque. This is a case of a 39-year-old female presenting with a three-month history of pruritic skin lesion over the right side of her trunk. On dermatological examination, there were large, discrete, band-like, hyperpigmented, papular patches following Blaschko’s lines on the right side of the trunk and abdomen. The histological examination of a biopsy taken from the lesion showed hypergranulosis, sawtooth rete ridges, band-like inflammatory infiltrate, confirming the diagnosis of lichen planus and was treated with topical steroids. Based on the findings, a planer, pruritic skin rash that follows Blaschko’s lines distribution rather than dermatomal distribution should raise the suspicion of zosteriform lichen planus.

## Introduction

Lichen planus (LP) has many clinical variants based on the morphology, site, and configuration of the lesion, including linear and zosteriform types [1]. Several cases have reported cutaneous LP but few of them had an incident with zosteriform LP that was reported [1]. In addition to LP, differential diagnoses of pruritic and pigmented skin lesion include linear psoriasis, lichen striatus, erythema dyschromicum, linear nevi, macular amyloidosis and post-inflammatory hyperpigmentation [2]. The definitive diagnosis is achieved by a histopathological examination of a biopsied lesion. One of the characteristic features of zosteriform LP is its distribution that is characterized by an eruption that follows Blaschko’s lines but is not restricted to a single dermatome [3].

## Case presentation

**Figure 1:**
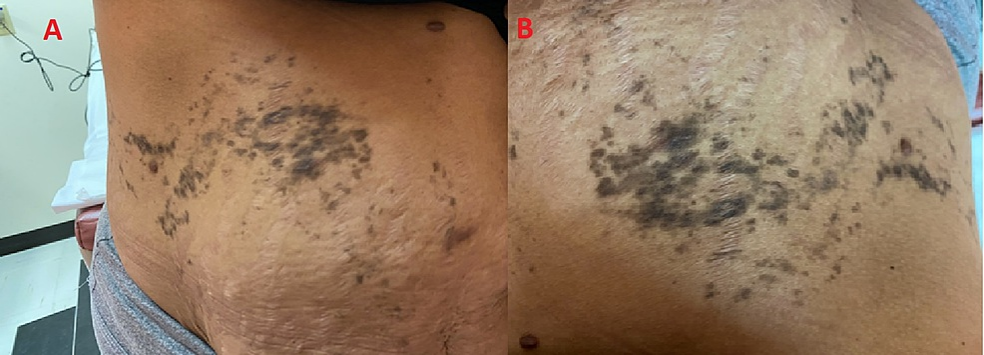
A. V-shaped configuration of lesions along the right side of the trunk, extending to the abdomen. B. S-shaped pattern of papular eruption along the right side of the abdomen.

A 39-year-old female presented to our dermatology clinic with a three-month history of moderate pruritic, hyperpigmented patches over the right side of her trunk. There was no previous history of any systemic or dermatological diseases such as herpes zoster. Additionally, the patient denied any family history or the use of any medication. Moreover, no prior consult was sought, and no intervention was previously done. Regarding her dermatological examination, it revealed multiple, hyperpigmented papules coalescing together forming a band-like shape over the right side of the trunk and abdomen. The distribution of the lesion was not restricted to a single dermatome; rather, it was found to follow Blaschko's lines (Figure 1) while no koebnerization was present and there was no scalp, oral, or genital mucosal involvement.

**Figure 2:**
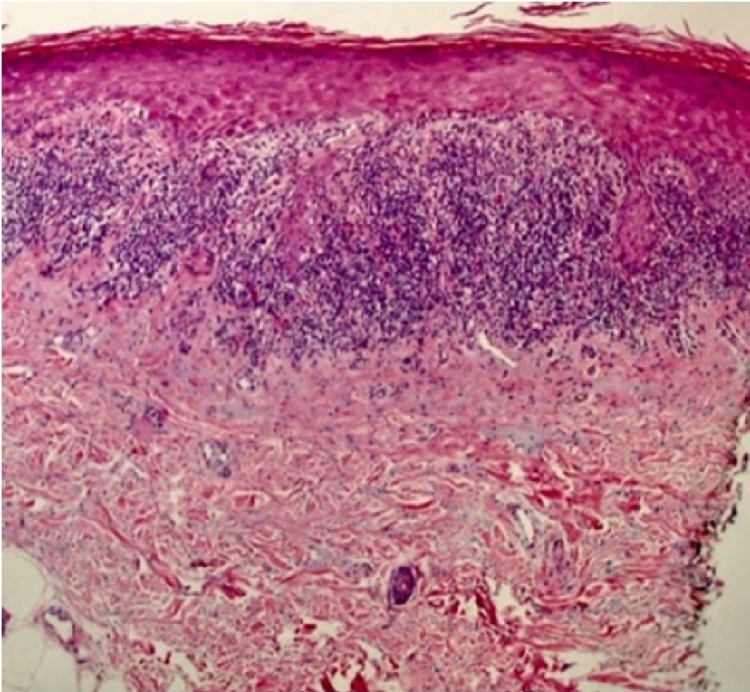
Histological findings of a bunch biopsy (H&E at x10): there are two major pathological findings in lichen planus; basal epidermal keratinocyte damage and an interface lymphocytic reaction.

As a result, the initial list of differential diagnoses included lichen planus, erythema dyschromicum, linear epidermal nevus, macular amyloidosis, post-inflammatory hyperpigmentation. In order to confirm the diagnosis, a punch biopsy was taken from the lesion for a histopathological examination reporting the presence of hypergranulosis, sawtooth rete ridges and a band-like inflammatory infiltrates (Figure 2).

A clinicopathological correlation was used for reaching the final diagnosis of zosteriform lichen planus. The patient was discharged home after setting a treatment plan with topical steroids of three months which was met with good compliance and experienced no adverse effects. A three-month follow-up showed a substantial improvement in the pruritus and a marked decrease in the hyperpigmentation but still persistent. The patient was happy after having her ailment finally diagnosed and was much happier to see the symptoms gradually regress. However, after two months, the follow-up visit was missed due to COVID-19 restrictions.

## Discussion

LP, described in 1892 by Kaposi, is a linear dermatosis with unique histological features affecting different sites of the body such as the skin, mucous membrane, hair follicle, and nails [4]. It is widespread throughout the world with no regard to race or sex [5] with incidence rates in males and females of 0.3% and 0.1%, respectively [2]. The exact etiology underlying LP is unknown; however, there is much evidence indicating the implication of an immune-mediated process in the pathophysiology of the disease [6]. Even though LP involves mainly the skin and mucus membrane, the basal layer of the epidermis that consists of keratinocytes and melanocytes is attacked by cytotoxic T cells; consequently, these cells are destroyed. As a result of this destruction, the melanin pigment leaks from the melanocytes causing hyperpigmentation of the skin [7].

LP has a moss-like appearance on the skin characterized by the well-known 5 Ps; purple, pruritic, polygonal, planer, papular rash [8]. Histologically, LP is characterized by the presence of wedge-shaped hypergranulosis, sawtooth rete ridges, as well as a band-like lymphocytic infiltrate beneath the epidermis that extends to the superficial layers causing a vacuolar change in the dermo-epidermal junction [7]. On the mucus membrane, LP is characterized by the presence of Wickham's striae, yet there was no mucus membrane involvement in our case. Since its discovery, there have been more than 20 clinical variants identified for LP based on the morphology, site, and configuration of the lesion [9]. Out of those variants, there are two similar types; zosteriform LP, which accounts for 0.2% of lichen planus cases, and linear LP. Nonetheless, they can be differentiated on dermatological examination [9].

Koebnerization is present in linear LP that leads to developing a linear pattern secondary to trauma that appears as narrow lines less than 2 cm in diameter following the course of the vein, or lymph vessel [9]. Whereas zosteriform LP has a band-like distribution consisting of wider lines more than 2 cm in diameter [7]. Moreover, they can be differentiated immunohistochemically through the detection of varicella-zoster antigens in the lesion of zosteriform LP; these antigens are not detected in linear LP [2]. Zosteriform LP is a rare clinical entity evolving spontaneously or as a result of previous herpes zoster. Spontaneous zosteriform LP does not follow specific dermatomal distribution. Instead, it follows Blaschko’s lines, which represent the migration of embryonic cells and do not correspond to the underlying structures of the skin such as nerves, muscles, and soft tissues [2]. Blaschko’s lines have specific superficial patterns on the upper spine (V-shape), the abdomen (S-shape), the breast area to the upper arm (inverted U-shape), and the front and back of the lower extremities (perpendicular to the afore-mentioned shapes). In our patient, the lesions followed the V-shaped pattern on the trunk and the S-shaped pattern on the abdomen (Figure 2). The treatment modality for all LP variants is the same. Whether the final diagnosis is zosteriform LP or linear LP, the preferred treatment for both types is topical steroids. Additionally, topical salicylic acid, topical tacrolimus, and systemic antihistamines can be used. The disease does not impede the normal activities of the patient; however, as the disease usually demonstrates an isomorphic response (Koebner phenomenon), the patient is advised to avoid any trauma to the skin.

## Conclusions

Zosteriform LP is a rare variant of LP with a characteristic distribution on the skin; however, the treatment modality is the same for all variants of LP. Zosteriform LP has the same histological features as LP, which distinguish it from other linear skin dermatoses such as linear psoriasis, linear nevi, and lichen striatus.
